# Investigation of Carbapenemase Resistance Genes and Plasmid-Mediated Quinolone Resistance Genes in Ceftolozane/Tazobactam-Resistant *Enterobacterales* and *Pseudomonas aeruginosa* Isolates

**DOI:** 10.3390/biomedicines14102224

**Published:** 2026-10-01

**Authors:** Canberk Çınar, Yeliz Tanrıverdi Çaycı, İlknur Bıyık, Ayşegül Çolak, Demet Gür Vural, Kemal Bilgin, Asuman Birinci

**Affiliations:** Department of Medical Microbiology, Faculty of Medicine, Ondokuz Mayıs University, Samsun 55139, Turkey; yeliztanriverdi@gmail.com (Y.T.Ç.); ilknurbiyik1@hotmail.com (İ.B.); aysegulcolaak@gmail.com (A.Ç.); demet-gur@yandex.com (D.G.V.); kemal.bilgin@omu.edu.tr (K.B.); asumanbirinci@yahoo.com (A.B.)

**Keywords:** ceftolozane–tazobactam, carbapenem-resistant *Enterobacterales*, *Pseudomonas aeruginosa*, carbapenemase genes, plasmid-mediated quinolone resistance

## Abstract

**Objectives:** Carbapenem-resistant Gram-negative bacteria limit treatment options for hospital-acquired infections, and resistance to ceftolozane/tazobactam is increasingly reported. This study investigated the distribution of carbapenemase and plasmid-mediated quinolone resistance (PMQR) genes among ceftolozane/tazobactam-resistant *Enterobacterales* and *Pseudomonas aeruginosa* isolates. **Methods:** We analyzed 224 non-duplicate ceftolozane/tazobactam-resistant clinical isolates (145 *Enterobacterales* and 79 *P. aeruginosa*) collected at Ondokuz Mayıs University Faculty of Medicine Hospital between January 2024 and March 2025. Species were identified by MALDI-TOF MS, and minimum inhibitory concentrations were determined with the BD Phoenix system and interpreted according to EUCAST breakpoints (v15.0; resistant: >2 mg/L for Enterobacterales, >4 mg/L for *P. aeruginosa*). Carbapenemase (*bla_OXA-48_*, *bla_NDM_*, *bla_KPC_*, *bla_VIM_*, *bla_IMP_*) and PMQR (*qnrA*, *qnrB*, *qnrC*, *qnrS*) genes were detected by multiplex PCR. **Results:** Among *Enterobacterales* (82.8% *Klebsiella pneumoniae*), *bla_OXA-48_* was the most frequent carbapenemase gene (50.3%), followed by *bla_NDM_* (25.5%) and *bla_KPC_* (17.2%); 24.8% of isolates carried none of the tested genes. *qnrB* was the most common PMQR gene (27.6%). Among *P. aeruginosa*, *bla_VIM_* (34.2%) and *bla_NDM_* (15.2%) were detected, and 51.9% of isolates were negative for all tested carbapenemase genes. PMQR genes were rare in *P. aeruginosa* (*qnrB*, 2.5%). *bla_IMP_* and *qnrC* were not detected in either group. **Conclusions:** Carbapenemase genes, mainly *bla_OXA-48_* in *Enterobacterales* and *bla_VIM_* in *P. aeruginosa*, were common among ceftolozane/tazobactam-resistant isolates, but many isolates were negative for all tested genes, suggesting that other mechanisms are involved. Because only resistant isolates were studied, these findings describe gene distribution and do not establish its contribution to resistance. Continued molecular surveillance is needed to support local treatment and infection control decisions.

## 1. Introduction

Antimicrobial resistance (AMR) is a major threat to global public health. Rising resistance rates, particularly among Gram-negative bacilli, complicate clinical management and are associated with increased morbidity and mortality in healthcare settings [1]. Infections caused by multidrug-resistant (MDR) and extensively drug-resistant (XDR) Gram-negative pathogens are especially challenging in intensive care units and in patients with severe underlying comorbidities. The slow pace of new antibiotic development, together with the rapid adaptation of bacteria, makes close monitoring of local resistance profiles and careful stewardship of existing agents essential [2]. Carbapenems have long been a main treatment option for severe infections caused by extended-spectrum β-lactamase (ESBL)-producing Gram-negative bacteria. However, the widespread dissemination of ESBL-producing Enterobacterales has led to increased carbapenem use, which in turn has contributed to the emergence and spread of carbapenem resistance. A major mechanism of carbapenem resistance is the production of carbapenemases, enzymes that hydrolyze carbapenems. Other mechanisms, such as reduced membrane permeability, altered β-lactamase activity, and structural mutations, may also contribute [3]. Carbapenemase genes, particularly *bla_OXA-48_*-like, *bla_NDM_*, *bla_KPC_*, *bla_VIM_*, and *bla_IMP_*, are frequently detected in clinically important Gram-negative pathogens, including *Enterobacterales* and *Pseudomonas aeruginosa*, and severely limit treatment options [4]. Plasmid-mediated quinolone resistance (PMQR) determinants, including *qnrA*, *qnrB*, *qnrC*, and *qnrS*, confer low-level reduced susceptibility to fluoroquinolones and facilitate horizontal transfer of resistance between bacterial species. Although PMQR genes alone usually cause only modest increases in minimum inhibitory concentrations (MICs), they often co-occur with other resistance determinants and promote the selection of high-level resistance, further narrowing treatment options [5]. Ceftolozane/tazobactam, an antipseudomonal cephalosporin combined with a β-lactamase inhibitor, is an important option for treating difficult-to-treat Gram-negative infections, particularly those caused by resistant *P. aeruginosa* [6]. However, increasing resistance to this agent is a growing clinical concern, and the molecular mechanisms behind it need to be clarified. Therefore, this study aimed to investigate the distribution of major carbapenemase genes (*bla_OXA-48_*, *bla_NDM_*, *bla_KPC_*, *bla_VIM_*, *bla_IMP_*) and PMQR genes (*qnrA*, *qnrB*, *qnrC*, *qnrS*) by multiplex PCR in ceftolozane/tazobactam-resistant clinical *Enterobacterales* and *P. aeruginosa* isolates, in order to provide epidemiological data on regional resistance patterns.

## 2. Materials and Methods

This retrospective cross-sectional study was approved by the Clinical Research Ethics Committee of Ondokuz Mayıs University Faculty of Medicine (26 August 2026; decision no. 2026/473). Ceftolozane/tazobactam-resistant Enterobacterales and *P. aeruginosa* isolates recovered from clinical specimens submitted to the Medical Microbiology Laboratory of Ondokuz Mayıs University Faculty of Medicine Hospital between January 2024 and March 2025 were retrieved from the laboratory’s frozen isolate collection (stored at −80 °C). To avoid overestimating gene prevalence, only one isolate per patient [the first ceftolozane/tazobactam-resistant isolate] was included, regardless of specimen type. In total, 224 isolates (145 *Enterobacterales* and 79 *P. aeruginosa*) were analyzed. Molecular analyses were performed on the archived isolates after ethics approval was obtained. Species-level identification was performed using matrix-assisted laser desorption/ionization time-of-flight mass spectrometry (MALDI-TOF MS) via the MALDI Biotyper Sirius system (Bruker Daltonics, Bremen, Germany). To avoid artificial inflation of gene prevalence rates and prevent epidemiological bias, strict patient-level deduplication criteria were applied: only a single non-duplicate clinical isolate per patient (the first isolated strain during the study period) was included in the final analysis, regardless of specimen type or repeated isolation. Antimicrobial susceptibility testing and minimum inhibitory concentration (MIC) determinations were performed using the BD Phoenix automated identification and susceptibility testing system (Becton Dickinson, Sparks, MD, USA). Antimicrobial susceptibility results and phenotypic resistance categories were interpreted strictly according to the European Committee on Antimicrobial Susceptibility Testing (EUCAST) clinical breakpoints (v15.0, 2025) [7]. Isolates exhibiting MIC values > 2 mg/L for *Enterobacterales* and >4 mg/L for *P. aeruginosa* were defined as resistant to ceftolozane/tazobactam. Bacterial genomic DNA was extracted using the boiling lysis method as previously described. Briefly, bacterial colonies from fresh 24 h cultures grown on Mueller–Hinton agar at 35 °C were suspended in 500 µL of sterile distilled water and homogenized by vortexing. The suspensions were incubated at 100 °C for 15 min on a dry heating block (Bioer, Hangzhou, China) for thermal cell lysis and subsequently centrifuged at 15,000× *g* at 4 °C for 20 min using a refrigerated centrifuge (Kubota, Tokyo, Japan). The supernatant containing the template DNA was transferred into sterile DNase/RNase-free microcentrifuge tubes and stored at −20 °C until amplification. The presence of carbapenemase genes (*bla_OXA-48_*, *bla_NDM_*, *bla_KPC_*, *bla_VIM_*, and *bla_IMP_*) and PMQR genes (*qnrA*, *qnrB*, *qnrC*, and *qnrS*) was investigated by multiplex PCR using validated, previously published specific oligonucleotide primer pairs. The primer sequences and their corresponding amplicon lengths were as follows: for *qnrA*, forward 5′-ATTTCTCACGCCAGGATTTG-3′ and reverse 5′-GATCGGCAAAGGTTAGGTCA-3′ (516 bp); for *qnrB*, forward 5′-GATCGTGAAAGCCAGAAAGG-3′ and reverse 5′-ATGAGCAACGATGCCTGGTA-3′ (476 bp); for *qnrC*, forward 5′-GGGTTGTACATTTATTGAATCG-3′ and reverse 5′-CACCTACCCATTTATTTTCA-3′ (307 bp); for *qnrS*, forward 5′-GCAAGTTCATTGAACAGGGT-3′ and reverse 5′-TCTAAACCGTCGAGTTCGGCG-3′ (428 bp) [8]; for *bla_KPC_*, forward 5′-TGT CAC TGT ATC GCC GTC-3′ and reverse 5′-CTC AGT GCT CTA CAG AAA ACC-3′ (900 bp); for *bla_NDM_*, forward 5′-GCA GCT TGT CGG CCA TGC GGG C-3′ and reverse 5′-GGT CGC GAA GCT GAG CAC CGC AT-3′ (782 bp); for *bla_VIM_*, forward 5′-GTT TGG TCG CAT ATC GCA AC-3′ and reverse 5′-AAT GCG CAG CAC CAG GAT AG-3′ (389 bp); for *bla_OXA-48_*, forward 5′-GCG TGG TTA AGG ATG AAC AC-3′ and reverse 5′-CAT CAA GTT CAA CCC AAC CG-3′ (438 bp); and for *bla_IMP_*, forward 5′-GAA GGC GTT TAT GTT CAT AC-3′ and reverse 5′-GTAC GTT TCA AGA GTG ATG C-3′ (188 bp) [9,10,11]. PCR reactions were performed using a thermal cycler (SensoQuest, Göttingen, Germany) with high-quality reagents including dNTP mixes (Thermo Fisher Scientific, Waltham, MA, USA). Reaction parameters including concentrations of dNTPs, MgCl2, primers, Taq DNA polymerase, and annealing temperatures were optimized to ensure high analytical specificity. Amplified products were separated by agarose gel electrophoresis and visualized using a gel documentation and imaging system (Bio-Rad Laboratories, Hercules, CA, USA). For quality control, well-characterized, previously sequenced and PCR-confirmed positive control strains for each target gene and template-free negative controls (ddH2O) were systematically included in every reaction batch. Specific oligonucleotide primer pairs utilized to amplify targeted resistance determinants yielded expected amplicon lengths ranging from 188 bp to 900 bp for downstream gel electrophoresis and molecular characterization. The multiplex PCR reaction mixture for the amplification of carbapenemase genes was prepared in a total volume of 50 µL, containing 5 µL of 10× PCR buffer, 4 µL of 25 mM MgCl_2_, 0.5 µL of dNTP mix (10 mM), 1 µL of Taq DNA polymerase (5 U/µL), 1 µL of each forward and reverse primer (20 pmol) specific for *bla_KPC_*, *bla_NDM_*, *bla_VIM_*, *bla_OXA-48_*, and *bla_IMP_*, 2 µL of template DNA, and 27.5 µL of nuclease-free water. The thermal cycling protocol for carbapenemase genes consisted of an initial denaturation step at 95 °C for 5 min, followed by 35 cycles of denaturation at 95 °C for 45 s, primer annealing at 60 °C for 45 s, and extension at 72 °C for 1 min, concluding with a final extension step at 72 °C for 8 min. Similarly, the multiplex PCR reaction mixture for the amplification of qnr genes was prepared in a total volume of 50 µL, containing 5 µL of 10× PCR buffer, 4 µL of 25 mM MgCl_2_, 1 µL of dNTP mix (10 mM), 1 µL of Taq DNA polymerase (5 U/µL), 1 µL of each forward and reverse primer (20 pmol) targeting *qnrA, qnrB, qnrC,* and *qnrS*, 2 µL of template DNA, and 29 µL of nuclease-free water. The thermal cycling conditions for the amplification of qnr genes comprised an initial denaturation step at 95 °C for 10 min, followed by 35 cycles of denaturation at 95 °C for 1 min, primer annealing at 61 °C for 1 min, and extension at 72 °C for 1 min, culminating in a final extension step at 72 °C for 10 min.

### Statistical Analysis

Categorical variables, including the prevalence of individual carbapenemase and PMQR genes, were expressed as absolute frequencies and percentages (n, %). The distribution of each carbapenemase gene (*bla_VIM_*, *bla_NDM_*, *bla_OXA-48_*, *bla_KPC_*) and PMQR gene (*qnrA*, *qnrB*, *qnrS*) between *Enterobacterales* and *P. aeruginosa* isolates was compared using Pearson’s Chi-square (χ^2^) test, as expected cell counts exceeded 5 in all corresponding 2 × 2 contingency tables. The *bla_IMP_* and *qnrC* genes were excluded from statistical comparison, as neither gene was detected in either species group. To account for multiple comparisons across the seven tested genes, a Bonferroni-corrected significance threshold of *p* < 0.007 (0.05/7) was applied. Statistical analyses were performed using SPSS software (version 26.0, IBM Corp., Armonk, NY, USA).

## 3. Results

Although *Enterobacterales* represents a taxonomic order comprising diverse genera and species—predominantly represented by *Klebsiella pneumoniae*, *Escherichia coli*, and *Enterobacter* spp. in our ceftolozane/tazobactam-resistant cohort—they were evaluated collectively to provide a comprehensive overview of resistance determinants across high-priority Gram-negative bacilli, alongside the specific species *Pseudomonas aeruginosa*. A total of 224 ceftolozane/tazobactam-resistant isolates were included in the study, comprising 145 *Enterobacterales* and 79 *P. aeruginosa* isolates. The distribution of the *Enterobacterales* isolates according to specimen type is presented in Table 1.

As summarized in Table 1, the clinical *Enterobacterales* isolates evaluated in this study were recovered from a diverse range of clinical specimens, with tracheal aspirates (n=46, 31.72%) and peripheral blood samples (n=33, 22.76%) accounting for the majority of the isolates.

All 224 clinical isolates evaluated in this study exhibited high-level phenotypic resistance to ceftolozane/tazobactam based on EUCAST clinical breakpoints (v15.0, 2025). Among *Enterobacterales* isolates, ceftolozane/tazobactam MIC values ranged from 4 mg/L to >16 mg/L (MIC_50_ = 8 mg/L, MIC_90_ > 16 mg/L). Furthermore, phenotypic analysis of the *Enterobacterales* cohort (n = 145) revealed high resistance rates to key alternative agents: phenotypic resistance to meropenem and imipenem was detected in 86.90% (n = 126) and 76.55% (n = 111) of the isolates, respectively, while resistance to ciprofloxacin and levofloxacin reached 91.03% (n = 132) and 84.83% (n = 123), respectively. For *P. aeruginosa* isolates, MIC values ranged from 8 mg/L to >32 mg/L (MIC_50_ = 16 mg/L, MIC_90_ > 32 mg/L). Similarly, the *P. aeruginosa* cohort (n = 79) demonstrated high phenotypic resistance rates to carbapenems and fluoroquinolones, with resistance to imipenem, meropenem, ciprofloxacin, and levofloxacin observed in 82.28% (n = 65), 84.81% (n = 67), 84.81% (n = 67), and 83.54% (n = 66) of the isolates, respectively. Regarding *P. aeruginosa* isolates, the highest isolation rate was observed in internal medicine wards (43.0%, n = 34), followed by intensive care units (31.6%, n = 25), surgical wards (19.0%, n = 15), and pediatric wards (6.3%, n = 5). The distribution of *P. aeruginosa* isolates according to specimen types is presented in Table 2.

As detailed in Table 2, the clinical *Pseudomonas aeruginosa* isolates evaluated in this study were recovered from various clinical sources, with midstream urine samples (n, 29.1%) and tracheal aspirates (n=17, 21.5%) being the most frequent specimen types.

Analysis of the species distribution among the *Enterobacterales* isolates showed that *Klebsiella pneumoniae* (*K. pneumoniae*) was the predominant species, accounting for 82.76% (n = 120) of the isolates. This was followed by *Escherichia coli* (*E. coli*) (7.59%, n = 11), *Enterobacter hormaechei* (*E. hormaechei*) (4.14%, n = 6), *Proteus mirabilis* (*P. mirabilis*) (2.07%, n = 3), and *Enterobacter cloacae* (*E. cloacae*) (1.38%, n = 2). *Citrobacter freundii* (*C. freundii*), *Klebsiella aerogenes* (*K. aerogenes*), and *Hafnia alvei* (*H. alvei*) were each detected in 0.69% (n = 1) of the isolates. The distribution of the investigated carbapenemase genes among ceftolozane/tazobactam-resistant *Enterobacterales* and *P. aeruginosa* isolates is presented in Table 3.

As summarized in Table 3, molecular screening revealed distinct resistance gene distributions between the two groups; *bla_OXA-48_* and $bla_{NDM}$ (25.52%) were the predominant carbapenemase genes detected among *Enterobacterales*, whereas $bla_{VIM}$ (34.18%) and $bla_{NDM}$ (15.19%) were the most frequently identified variants in *P. aeruginosa*.

Among the *Enterobacterales* isolates, the most frequently detected carbapenemase gene was *bla_OXA-48_* (50.34%, n = 73), followed by *bla_NDM_* (25.52%, n = 37), *bla_KPC_* (17.24%, n = 25), and *bla_VIM_* (6.21%, n = 9). The *bla_IMP_* gene was not detected in any of the *Enterobacterales* isolates. Among the *P. aeruginosa* isolates, *bla_VIM_* was the most frequently detected carbapenemase gene (34.18%, n = 27), followed by *bla_NDM_* (15.19%, n = 12). The *bla_OXA-48_*, *bla_KPC_*, and *bla_IMP_* genes were not detected in any of the *P. aeruginosa* isolates. Comparative analysis revealed that the frequency of *bla_OXA-48_* (χ^2^(1, N = 224) = 59.00, *p* < 0.001), *bla_VIM_* (χ^2^(1, N = 224) = 29.66, *p* < 0.001), and *bla_KPC_* (χ^2^(1, N = 224) = 15.33, *p* < 0.001) differed significantly between *Enterobacterales* and *P. aeruginosa* isolates, remaining significant after Bonferroni correction (*p* < 0.007); in contrast, *bla_NDM_* did not differ significantly between the two species (χ^2^(1, N = 224) = 3.19, *p* = 0.074). Regarding PMQR genes, *qnrB* was the most frequently detected gene among the *Enterobacterales* isolates (27.59%, n = 40), followed by *qnrS* (14.48%, n = 21) and *qnrA* (12.41%, n = 18). The *qnrC* gene was not detected in any of the *Enterobacterales* isolates. Among the *P. aeruginosa* isolates, *qnrB* was detected in only two isolates (2.53%), whereas *qnrA*, *qnrC*, and *qnrS* were not detected. The distribution of *qnrA* (χ^2^(1, N = 224) = 10.66, *p* = 0.001), *qnrB* (χ^2^(1, N = 224) = 21.07, *p* < 0.001), and *qnrS* (χ^2^(1, N = 224) = 12.62, *p* < 0.001) also differed significantly between the two species, with all remaining significant after Bonferroni correction. The positivity rates of the investigated PMQR genes are presented in Table 4.

As outlined in Table 4, plasmid-mediated quinolone resistance genes were predominantly detected among the *Enterobacterales* isolates, with qnrB (27.59%) and qnrS (14.48%) being the most prevalent variants, whereas only a minor proportion of *P. aeruginosa* isolates harbored qnrB (2.53%).

As *K. pneumoniae* constituted the vast majority of the *Enterobacterales* cohort (n = 120, 82.76%), species-specific resistance gene distributions were further evaluated for this predominant pathogen. Among *K. pneumoniae* isolates, *bla_OXA-48_* was the most frequently detected carbapenemase gene (53.33%, n = 64), followed by *bla_NDM_* (28.33%, n = 34), *bla_KPC_* (19.17%, n = 23), and *bla_VIM_* (6.67%, n = 8), while *bla_IMP_* was not detected. Regarding plasmid-mediated quinolone resistance determinants within the *K. pneumoniae* group, *qnrB* was the most prevalent variant (29.17%, n = 35), followed by *qnrS* (16.67%, n = 20) and *qnrA* (13.33%, n = 16), with *qnrC* remaining entirely absent. In *Enterobacterales* isolates, the most frequently detected carbapenemase gene profile was *bla_OXA-48_* positivity alone, followed by co-positivity for *bla_OXA-48_* and *bla_NDM_*, and *bla_KPC_* positivity alone. Less frequently, gene combinations including *bla_VIM_*, *bla_OXA-48_*+*bla_KPC_*, *bla_VIM_*+*bla_OXA-48_*, *bla_VIM_*+*bla_NDM_*, *bla_OXA-48_*+*bla_NDM_*+*bla_KPC_*, *bla_VIM_*+*bla_OXA-48_*+*bla_NDM_*, and *bla_OXA-48_*+*bla_VIM_*+*bla_KPC_* were detected, while a group of isolates was negative for the tested carbapenemase genes. The positivity rates of the detected carbapenemase genes are given in Table 5. In *P. aeruginosa* isolates, *bla_VIM_* positivity was most commonly identified, followed by *bla_NDM_* positivity; only a single isolate was co-positive for *bla_VIM_* and *bla_NDM_*, while the remaining isolates were negative for the tested carbapenemase genes. The carbapenemase genes detected in *P. aeruginosa* isolates are presented in Table 6.

As detailed in Table 5, while a quarter of the *Enterobacterales* isolates (24.83%) tested negative for the investigated carbapenemase genes, the remaining isolates exhibited diverse single and co-carriage profiles, with sole *bla_OXA-48_* (28.97%) and the dual combination of *bla_OXA-48_* and $bla_{NDM}$ (16.55%) being the most frequent patterns.

As summarized in Table 6, more than half of the *P. aeruginosa* isolates (51.9%) were negative for the targeted carbapenemase genes, whereas positive isolates predominantly harbored single genes, specifically $bla_{VIM}$ (32.9%) and $bla_{NDM}$ (13.9%), alongside a rare co-carriage instance of $bla_{VIM}$ and $bla_{NDM}$ (1.3%).

## 4. Discussion

The introduction of antibiotics has led to major advances in the treatment of infectious diseases; however, the growing threat of antimicrobial resistance is now jeopardizing these achievements. In particular, the increasing prevalence of mechanisms such as ESBL production, carbapenem resistance, and multidrug resistance among hospital-acquired infections caused by Gram-negative bacteria has significantly limited the effectiveness of available treatment options. Rising resistance rates among important nosocomial pathogens, including *Escherichia coli*, *Klebsiella pneumoniae*, *Pseudomonas aeruginosa*, and *Acinetobacter* spp., have made the management of invasive infections increasingly challenging. The fact that the development of new antibacterial agents is lagging behind the emergence of antimicrobial resistance has made preserving effective treatment options and developing novel antimicrobial strategies a global health priority [12]. In clinical presentations caused by strains with MDR and XDR phenotypes, delay or an inappropriate selection of empirical antimicrobial regimens prolongs hospital stays, increases treatment costs, and correlates with significantly high morbidity and mortality rates [13]. Indeed, the World Health Organization (WHO) has highlighted carbapenem-resistant *Enterobacterales* among critical priority pathogens and *P. aeruginosa* among high-priority pathogens requiring urgent research and the development of new therapeutic molecules [14]. In this context, the determination of transposon- or plasmid-borne horizontal resistance transfer dynamics and molecular epidemiological profiles is of critical importance for designing rational antibiotic use strategies and structuring infection control protocols [15]. *Enterobacterales* and *P. aeruginosa* are among the primary etiological agents of healthcare-associated infections (HAIs), and the extended-spectrum resistance patterns observed in these microorganisms dramatically limit the chances of clinical success [16]. In this study, the genotypic distribution of carbapenemase genes and PMQR markers in clinical *Enterobacterales* and *P. aeruginosa* isolates exhibiting phenotypic resistance to ceftolozane/tazobactam was evaluated at the molecular level. In the genotypic analysis of our study, the *bla_OXA-48_* gene was identified as the most predominant resistance gene among the *Enterobacterales* isolates, followed by *bla_NDM_* and *bla_KPC_* genes, respectively. The fact that *bla_OXA-48_*, a Class D carbapenemase, has a high endemic prevalence in Turkey and dominates the regional gene pool shows full agreement with large-scale molecular epidemiology data in the literature [17,18]. Furthermore, the detection of *bla_OXA-48_* and *bla_NDM_* detected in our study demonstrates the current prevalence of beta-lactamase hydrolysis capacity in our geographical region. Moreover, the detection of *bla_NDM_* (encoding a metallo-beta-lactamase [MBL]) and *bla_KPC_* (encoding a Class A carbapenemase) at relatively comparable frequencies in our isolates highlights the diverse distribution of carbapenemase determinants and reflects regional epidemiological patterns [19]. On the other hand, in the *P. aeruginosa* isolates included in the study, it was confirmed that the dominant detected genotypic resistance factors were *bla_VIM_* and *bla_NDM_* genes, which fall into the MBL class. While enzymatic inactivation by MBLs constitutes a highly potent resistance mechanism with significant potential for mobile dissemination as reported in the literature, it is crucial to recognize that ceftolozane/tazobactam resistance—particularly in *P. aeruginosa*—is frequently multifactorial, involving chromosomal adaptations such as outer membrane porin alterations or efflux pump overexpression alongside acquired determinants. Therefore, the detection of MBL determinants carried by mobile genetic elements provides vital insight into local resistance mechanisms, yet it must be interpreted alongside potential chromosomal pathways. The combination of ceftolozane, a novel extended-spectrum antipseudomonal cephalosporin, with tazobactam, a potent beta-lactamase inhibitor, is recognized as a vital option in the treatment of multidrug-resistant Gram-negative pathogens [20]. In our study, ceftolozane/tazobactam-resistant isolates revealed the presence of multiple carbapenemase genes, indicating that resistant strains frequently harbor these specific molecular determinants rather than implying a direct causal pathway or a single mechanism. Although resistance to quinolone antibiotics is primarily driven by chromosomal point mutations in the DNA gyrase (*gyrA*) and Topoisomerase IV (*parC*) gene regions [21], PMQR mechanisms demand high clinical vigilance due to their association with extrachromosomal resistance traits. In our data, *qnrB* was the predominant PMQR determinant among *Enterobacterales*, indicating its notable contribution to the observed plasmid-mediated quinolone resistance profile. The rapid dissemination of carbapenemase-producing strains within the nosocomial ecosystem is considered one of the most critical drivers of the global pan-resistance crisis [22]. Recent international surveillance studies have reported that ceftolozane/tazobactam resistance involves both chromosomal adaptation and acquired carbapenemases spreading via mobile genetic elements [23]. The notable presence of *bla_VIM_* and *bla_NDM_* detected in *P. aeruginosa* isolates in our research findings parallels Eurasian surveillance data highlighting the heavy enzymatic burden on antipseudomonal agents [24]. Moreover, the observed co-positivity of *bla_OXA-48_* and *bla_NDM_* among *Enterobacterales* highlights the complexity of carbapenemase-mediated resistance in high-risk hospital settings [25].

### Study Limitations

This study has several limitations. First, ceftolozane/tazobactam MICs were determined only with the BD Phoenix automated system and were not confirmed by broth microdilution, the EUCAST reference method. Isolates with MICs close to the breakpoint (the lowest MIC observed in *Enterobacterales* was 4 mg/L, against a resistance breakpoint of >2 mg/L) may therefore have been misclassified. Second, we included only ceftolozane/tazobactam-resistant isolates and had no susceptible comparison group, so we cannot assess whether the detected genes are associated with resistance. Isolate-level analysis of genotype and phenotype, as well as clinical data, was not performed. Third, only the selected carbapenemase (*bla_OXA-48_*, *bla_NDM_*, *bla_KPC_*, *bla_VIM_*, *and bla_IMP_*) and PMQR (*qnrA*, *qnrB*, *qnrC*, *qnrS*) genes were screened by multiplex PCR. Other carbapenemases and β-lactamases (e.g., *bla_GES_*, *bla_PER_*), other PMQR determinants (*qnrD*, *qepA*, *aac(6′)-Ib-cr*), and OXA-48-like variants could not be identified, as PCR products were not sequenced. Fourth, chromosomal mechanisms relevant to ceftolozane/tazobactam resistance, such as AmpC overproduction or structural modification, PBP3 mutations, efflux pump overexpression, and porin alterations, were not investigated and may explain resistance in the PCR-negative isolates, particularly in *P. aeruginosa*. Fifth, phenotypic carbapenemase assays (e.g., mCIM/eCIM), gene expression analysis, and molecular typing were not performed, so we cannot determine whether the detected genes were functional or whether clonal spread contributed to their distribution. Finally, *Enterobacterales* were analyzed together, although most isolates (82.8%) were *K. pneumoniae*, and the study was conducted at a single tertiary-care center, which limits generalizability. Multicenter studies including susceptible isolates, whole-genome sequencing, and gene expression analysis are needed to confirm these findings.

## 5. Conclusions

Among ceftolozane/tazobactam-resistant isolates, *bla_OXA-48_* and *bla_NDM_* were the most frequent carbapenemase genes in *Enterobacterales*, predominantly *K. pneumoniae*, whereas *bla_VIM_* and *bla_NDM_* were detected in *P. aeruginosa*. However, 24.8% of *Enterobacterales* and 51.9% of *P. aeruginosa* isolates carried none of the tested carbapenemase genes, indicating that other mechanisms are likely involved. PMQR genes, mainly *qnrB*, were common in *Enterobacterales* but rare in *P. aeruginosa*. Since only resistant isolates were included and gene expression, genetic context, and chromosomal mechanisms were not evaluated, these findings describe the local distribution of the tested genes and do not establish their contribution to ceftolozane/tazobactam resistance. Continued molecular surveillance, ideally with whole-genome sequencing and a susceptible comparison group, is warranted to guide empirical therapy and infection control.

## Figures and Tables

**Table 1:** Distribution of *Enterobacterales* isolates according to specimen types.

Specimen Type	Number (n)	Percentage (%)
Tracheal aspirate	46	31.72%
Peripheral blood	33	22.76%
Catheter hub blood sample	19	13.10%
Catheter tip	15	10.34%
Blood culture	9	6.21%
Wound swab	9	6.21%
Surgical tissue/specimen	7	4.83%
Abscess aspirate/sample	3	2.07%
Cerebrospinal fluid (CSF)	2	1.38%
Sputum	1	0.69%
Body fluid	1	0.69%

**Table 2:** Distribution of *P. aeruginosa* isolates according to specimen types.

Specimen Type	Number (n)	Percentage (%)
Midstream urine	23	29.1%
Tracheal aspirate	17	21.5%
Surgical specimens/tissue	10	12.7%
Wound swabs	6	7.6%
Blood sample	5	6.3%
Catheter hub blood sample	5	6.3%
Sputum	4	5.1%
Abscess	3	3.8%
Peripheral blood sample	2	2.5%
Ear swab	2	2.5%
Bronchoalveolar lavage (BAL)	1	1.3%
Body fluid	1	1.3%

**Table 3:** Distribution of carbapenemase resistance genes (*bla_KPC_*, *bla_NDM_*, *bla_VIM_*, *bla_IMP_*, *bla_OXA-48_*).

Species/Group	*bla_VIM_*	*bla_NDM_*	*bla_OXA-48_*	*bla_KPC_*	*bla_IMP_*
*Enterobacterales*	9 (6.21%)	37 (25.52%)	73 (50.34%)	25 (17.24%)	0 (0%)
*P. aeruginosa*	27 (34.18%)	12 (15.19%)	0 (0%)	0 (0%)	0 (0%)

**Table 4:** Distribution of plasmid-mediated quinolone (*qnrA, qnrB, qnrC*, and *qnrS*) resistance genes.

Microorganism	*qnrA*	*qnrB*	*qnrC*	*qnrS*
*Enterobacterales*	18 (12.41%)	40 (27.59%)	0 (0%)	21 (14.48%)
*P. aeruginosa*	0 (0%)	2 (2.53%)	0 (0%)	0 (0%)

**Table 5:** Carbapenemase gene rates in *Enterobacterales* isolates.

Gene Profile	n	%
Negative	36	24.83
*bla_OXA-48_*	42	28.97
*bla_OXA-48_*, *bla_NDM_*	24	16.55
*bla_KPC_*	21	14.48
*bla_NDM_*	10	6.90
*bla_VIM_*	4	2.76
*bla_OXA-48_*, *bla_KPC_*	2	1.38
*bla_VIM_*, *bla_OXA-48_*	2	1.38
*bla_VIM_*, *bla_NDM_*	1	0.69
*bla_VIM_*, *bla_OXA-48_*, *bla_NDM_*	1	0.69
*bla_OXA-48_*, *bla_NDM_*, *bla_KPC_*	1	0.69
*bla_OXA-48_*, *bla_VIM_*, *bla_KPC_*	1	0.69

**Table 6:** Carbapenemase gene rates in *P. aeruginosa* isolates.

Gene Profile	n	%
Negative	41	51.9
*bla_VIM_*	26	32.9
*bla_NDM_*	11	13.9
*bla_VIM_*, *bla_NDM_*	1	1.3

## Data Availability

The datasets generated and/or analyzed during the current study are available from the corresponding author upon reasonable request.
